# The effect of adenine protonation on RNA phosphodiester backbone bond cleavage elucidated by deaza-nucleobase modifications and mass spectrometry

**DOI:** 10.1093/nar/gkz574

**Published:** 2019-07-05

**Authors:** Elisabeth Fuchs, Christoph Falschlunger, Ronald Micura, Kathrin Breuker

**Affiliations:** Institute of Organic Chemistry and Center for Molecular Biosciences Innsbruck (CMBI), University of Innsbruck, Innrain 80/82, 6020 Innsbruck, Austria

## Abstract

The catalytic strategies of small self-cleaving ribozymes often involve interactions between nucleobases and the ribonucleic acid (RNA) backbone. Here we show that multiply protonated, gaseous RNA has an intrinsic preference for the formation of ionic hydrogen bonds between adenine protonated at N3 and the phosphodiester backbone moiety on its 5′-side that facilitates preferential phosphodiester backbone bond cleavage upon vibrational excitation by low-energy collisionally activated dissociation. Removal of the basic N3 site by deaza-modification of adenine was found to abrogate preferential phosphodiester backbone bond cleavage. No such effects were observed for N1 or N7 of adenine. Importantly, we found that the pH of the solution used for generation of the multiply protonated, gaseous RNA ions by electrospray ionization affects phosphodiester backbone bond cleavage next to adenine, which implies that the protonation patterns in solution are at least in part preserved during and after transfer into the gas phase. Our study suggests that interactions between protonated adenine and phosphodiester moieties of RNA may play a more important mechanistic role in biological processes than considered until now.

## INTRODUCTION

**Scheme 1.**
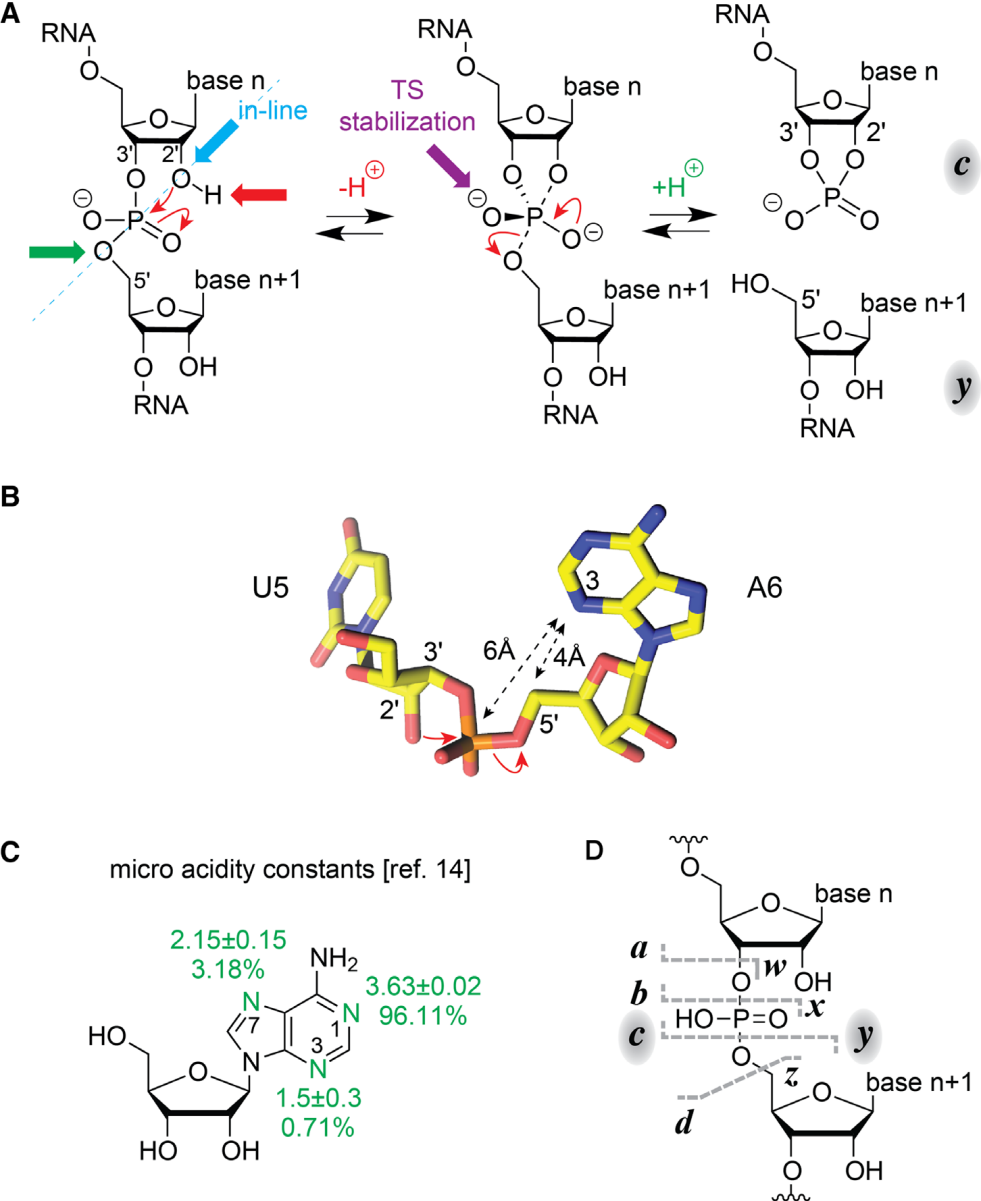
RNA phosphodiester cleavage by phosphoester transfer involving the 2′-hydroxyl group. (**A**) The internucleotide linkage (‘scissile’ phosphate) passes through a pentacoordinate transition state that results in a 2′,3′-cyclic phosphate (***c***) and a 5′-hydroxyl (***y***) cleavage product. The four catalytic strategies that can facilitate the reaction are: α, in-line nucleophilic attack, S_N_2-type (blue); β, neutralization of the (developing) negative charge on a non-bridging phosphate oxygen, i.e. transition state (TS) stabilization (purple); γ, deprotonation of the 2′-hydroxyl group (red); and δ, neutralization of the negative charge on the 5′-oxygen by protonation (green). This color code also matches the proton annotations above the reaction arrows. (**B**) The active site of the twister ribozyme holds A6 in *syn*-conformation (PDB code: 4RGE), which indicates that a direct (or water-mediated) interaction between N3 and the scissile phosphate between U5 and A6 can form after additional conformational changes (distances are highlighted by double arrows). For the twister ribozyme, it is known that atomic mutagenesis (N3 to C3) of A6 abolishes cleavage, which further hints at the relevance of this potential interaction (3,12). Together these features were inspiring for the present study that aims at revealing sequence-specific fragmentation of gaseous RNA in mass spectrometric experiments. (**C**) Micro acidity constants of adenosine and corresponding percentages of tautomers of monoprotonated adenosine present in aqueous solution as determined by Sigel and coworkers (14). (**D**) Mass spectrometry nomenclature for fragments from RNA backbone cleavage: CAD predominantly results in complementary ***c*** and ***y*** fragments, corresponding to the 2′,3′ cyclic phosphate and 5′-OH cleavage products shown in A (the dashed lines indicate possible cleavage sites without implying a specific mechanism).

Phosphodiester backbone bond cleavage of ribonucleic acids (RNA) is a fundamental chemical process in living systems. Cells transcribe far more RNA than they accumulate, and maintain tightly regulated degradation systems for cleavage of damaged or redundant RNA (1). The actual cleavage reaction is accomplished by ribonucleases (RNases). RNases fall into three classes, with endonucleases that cut RNA internally, 5′-exonucleases that hydrolyze RNA from the 5′-end and 3′-exonucleases that degrade RNA from the 3′-end (1). Apart from protein enzymes, RNA enzymes (referred to as ribozymes) can catalyze RNA phosphodiester backbone bond cleavage. Ribozymes are widely distributed in nature and involved in a number of essential biological processes including replication of RNA genomes and mobile genetic elements, RNA splicing, translation and RNA degradation (2). To date, nine distinct classes of small self-cleaving ribozymes are known, of which four—the twister, twister sister, pistol and hatchet ribozymes—were discovered only recently (3,4). All self-cleaving ribozymes fold into intricate three-dimensional structures with active sites that catalyze the site-specific cleavage of a single phosphodiester backbone bond (3,5). The catalytic strategies (5) of ribozymes for RNA cleavage into 2′,3′-cyclophosphate and 5′-OH cleavage products inevitably include pre-orientation of the 2′-OH nucleophile toward the (to-be-attacked) scissile phosphate (in-line attack, Scheme 1A). All other strategies rely on the proximity of charge or on charge transfer reactions (6), for example, site-specific metal ion cofactors such as Mg^2+^ for stabilization of the transition state or nucleobases with shifted p*K* values for general acid-base catalysis (3–5,7–11). Ribozymes usually apply a combination of strategies. For cleavage of the phosphodiester backbone bond between U5 and A6 of twister (Scheme 1B), the major contributions to catalysis stem from G48 (assisting in both 2′-OH activation and transition state stabilization) and from A6 directly at the scissile phosphate (12,13). The shifted p*K* value of A6 determined in nuclear magnetic resonance (NMR) spectroscopic experiments (13) suggests a crucial role of this nucleobase in general acid catalysis for stabilization of the O5′ leaving group through proton transfer (for adenosine micro acidity constants see Scheme 1C and reference (14)).

Unfolded, single-stranded RNA also degrades in solutions that do not contain appreciable concentrations of metal ions (15), and several studies in the literature have described sequence specificity for this process (16–19) that is commonly referred to as RNA hydrolysis (20). For example, Linjalahti and Mikkola found that at pH 5.5 and 6.5, the rate constants for hydrolysis of RNA phosphodiester backbone bonds between U and A can exceed those between U and U by a factor of ∼300 (19). At near-neutral pH at which the phosphodiester moiety and the 2′-OH group are deprotonated and uncharged, respectively, cleavage of the phosphodiester backbone bonds between pyrimidine and adenine seems favored in the hydrolysis of single-stranded short oligoribonucleotides (19,21,22). Interestingly, AU-rich elements substantially destabilize messenger RNA stability (23). Hydrolysis of ∼1100 nt homopolymers of A, C, G and U ribonucleotides at pH 8 and 50°C showed an order of stability poly(A) < poly(U) < poly(C) < poly(G) (24). Both sequence (21,22,25) and higher order structure (26,27) affect RNA hydrolysis, and although highly structured, small ribozymes cleave RNA with far higher specificity, the chemical structures of the 2′,3′-cyclophosphate and 5′-OH cleavage products from hydrolysis are the same.

In our laboratory, we develop mass spectrometry (MS) based approaches for RNA characterization that involve cleavage of multiply protonated or deprotonated RNA ions (15,28–34), more commonly referred to as ‘RNA dissociation’ in the mass spectrometry community (35). We have recently investigated the effect of ribose and backbone modifications on phosphodiester backbone bond cleavage in collisionally activated dissociation (CAD) of fully desolvated, gaseous (M+nH)^n+^ and (M-nH)^n−^ ions of RNA produced by electrospray ionization (ESI) (36). CAD is a commonly used method for incrementally increasing the vibrational energy of gaseous ions until fragmentation occurs. Low-energy CAD of RNA predominantly produces ***c*** and complementary ***y*** fragments (Scheme 1D) that correspond to 2′,3′ cyclic phosphate and 5′ OH cleavage products (Scheme 1A), respectively. Our previous study revealed preferred phosphodiester backbone bond cleavage on the 5′-side of adenosine in CAD of (M+nH)^n+^ ions of several oligoribonucleotides, for which we postulated a mechanism that is based on ionic hydrogen bonding between adenine protonated at N3 and the phosphodiester moiety on its 5′ side (36). Here we report a conclusive test for this hypothesis in a comprehensive study of short RNAs with and without 1-deaza-, 3-deaza- and 7-deazaadenosine modifications. The CAD data confirm our proposed mechanism as only 3-deazaadenosine, but not 1-deaza- or 7-deazaadenosine, interfere with preferred phosphodiester backbone bond cleavage in CAD of (M+nH)^n+^ ions. Strikingly, we observed a correlation between the site-specific extent of phosphodiester backbone bond cleavage and the pH of the solution used for ESI, indicating that the protonation pattern in solution is at least in part preserved in the gaseous RNA ions. Our study thus contributes to a deeper understanding of the intrinsic preference of adenosine to facilitate RNA phosphodiester cleavage in solution and in the gas phase.

## MATERIALS AND METHODS

Experiments were performed on a 7 T Fourier transform ion cyclotron resonance (FT-ICR) mass spectrometer (Bruker, Austria) equipped with an ESI source for (M+nH)^n+^ or (M-nH)^n−^ ion generation and a collision cell through which a flow of Ar gas was maintained for CAD. RNA was electrosprayed (flow rate 1.5 μl/min) from 0.5–1.0 μM solutions in 1:1 H_2_O/CH_3_OH with 20 mM ammonium acetate (for production of (M+nH)^n+^ ions) or 20 mM piperidine (for production of (M-nH)^n−^ ions) as additives, and polyethylene glycol 1000 (Sigma-Aldrich, Austria) was used as internal calibrant for accurate mass measurements (Table 1). In solutions with ammonium acetate, NH_4_^+^ replaces Na^+^ or K^+^ as counterion for the deprotonated phosphodiester moieties of nucleic acids, and during desolvation by ESI, NH_3_ readily dissociates while leaving protons as counterions (37–40) such that the (M+nH)^n+^ RNA ions produced by ESI in positive mode have uncharged phosphodiester moieties (36,41). Solution pH was measured using non-bleeding pH-indicator strips (Merck, Germany). Methanol was HPLC grade (Acros, Austria), H_2_O was purified to 18 MΩ·cm at room temperature using a Milli-Q system (Millipore, Austria), ammonium acetate (≥99.0%, Na ≤5 mg/kg, K ≤5 mg/kg) and piperidine (≥99.5%) were from Sigma-Aldrich (Austria). The (M+nH)^n+^ or (M-nH)^n−^ ions under study were isolated in a linear quadrupole prior to dissociation by CAD; for a more detailed description of the experimental setup, see reference (34). For statistical reasons, between 50 and 200 scans were added for each spectrum (50 for ESI, 100 for CAD of (M-nH)^n−^ ions, 200 for CAD of (M+nH)^n+^ ions), and data reduction utilized the SNAP2 algorithm (Bruker, Austria).

**Table 1.** RNA studied

RNA	Sequence^a^	M_measured_^b^	M_calculated_^b^
**1**	GAAGG GAAAC CUUCG	4835.718	4835.718
**2**	GAAGG GA**c^3^A**AC CUUCG	4834.721	4834.723
**3**	GAAGG GA**c^1,3^A**AC CUUCG	4833.734	4833.727
**4**	GAAGG GA**c^1^A**AC CUUCG	4834.728	4834.723
**5**	GAAGG GA**c^7^A**AC CUUCG	4834.718	4834.723
**6**	GAAGG GA**dA**AC CUUCG	4819.727	4819.724
**7**	GAAGG GCAAC CUUCG	4811.705	4811.707
**8**	GAAGG GC**c^3^A**AC CUUCG	4810.713	4810.712
**9**	GAAGG GCA**c^3^A**C CUUCG	4810.714	4810.712
**10**	GAAGG GC**c^1^A**AC CUUCG	4810.713	4810.712
**11**	GAAGG GCA**c^1^A**C CUUCG	4810.713	4810.712
**12**	GAAGG GC**c^7^A**AC CUUCG	4810.707	4810.712
**13**	GAAGG GCA**c^7^A**C CUUCG	4810.712	4810.712
**14**	GAAGG GCA**dA**C CUUCG	4795.715	4795.712

^a^from 5′-OH- to 3′-OH-terminus, ^b^in Da; M refers to monoisotopic mass

Nucleoside phosphoramidite building blocks of dA, A, C, G and U were purchased from ChemGenes (Wilmington, MA, USA), and those of 3-deazaadenosine (c^3^A), 1-deazaadenosine (c^1^A) and 1,3-dideazaadenosine (c^1,3^A) were synthesized as described in references (42) and (43). RNAs **1**–**14** (Table 1) were prepared by solid-phase synthesis (44,45) and purified by HPLC. For desalting, ∼400 μl of an ammonium acetate solution (100 mM in H_2_O) was added to ∼100 μl RNA solution (2.5–10 nmol in H_2_O) and concentrated to ∼50 μl using Vivaspin 500 centrifugal concentrators (Sartorius, Germany, PES membrane, MWCO 3000). The process was repeated six times, followed by six cycles of concentration and dilution with H_2_O, after which RNA concentration was determined by UV absorption at 260 nm using a NanoPhotometer (Implen, Germany). CAD of (M-nH)^n−^ ions was used to confirm the sequence of RNAs **1**–**14**.

## RESULTS AND DISCUSSION

**Figure 1.**
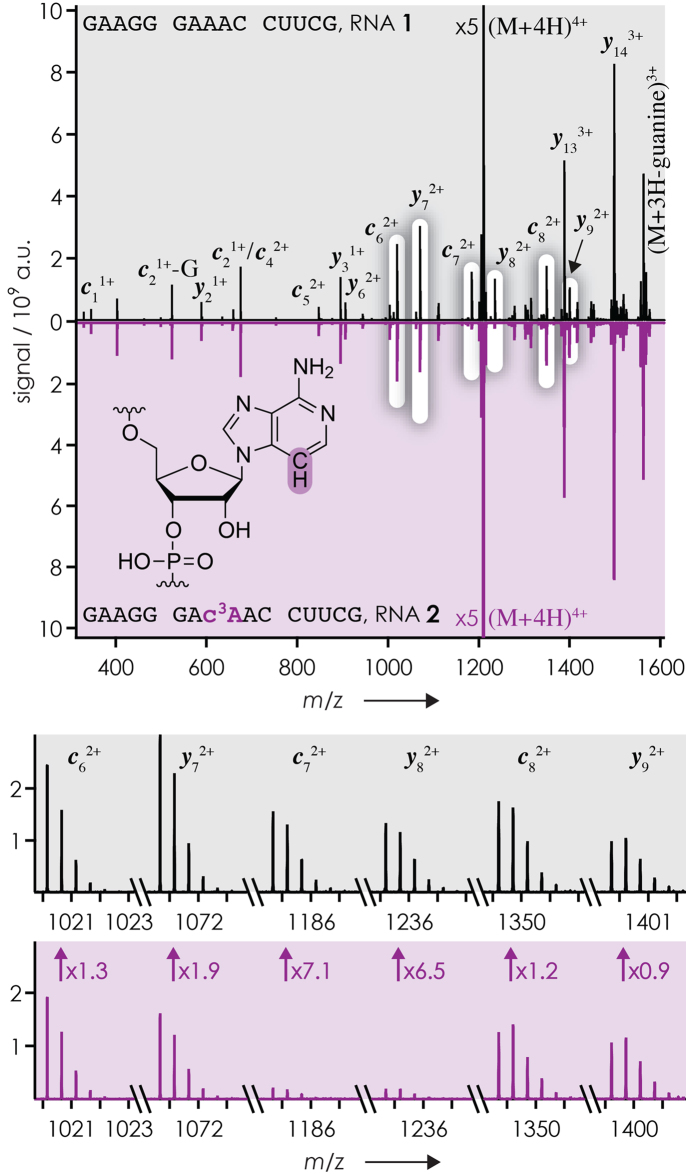
CAD spectra (42 eV laboratory frame collision energy) of (M+4H)^4+^ ions of RNA **1** (black) and **2** (purple, mirrored to facilitate comparison) electrosprayed from 1 μM solutions in 1:1 H_2_O/CH_3_OH with 20 mM ammonium acetate as additive (pH 6.8); signals of complementary ***c***_7_^2+^ and ***y***_8_^2+^ fragments from backbone cleavage between residues 7 and 8 (bottom; ***c***_6_^2+^, ***c***_8_^2+^, ***y***_7_^2+^, and ***y***_9_^2+^ shown for comparison) decreased by a factor of ∼7 as a result of replacing A with c^3^A at position 8; the difference in *m/z* of ***y***_8_^2+^, ***c***_8_^2+^, and ***y***_9_^2+^ from CAD of RNAs **1** and **2** reflects the mass difference between A and c^3^A of 0.9953 Da.

**Figure 2.**
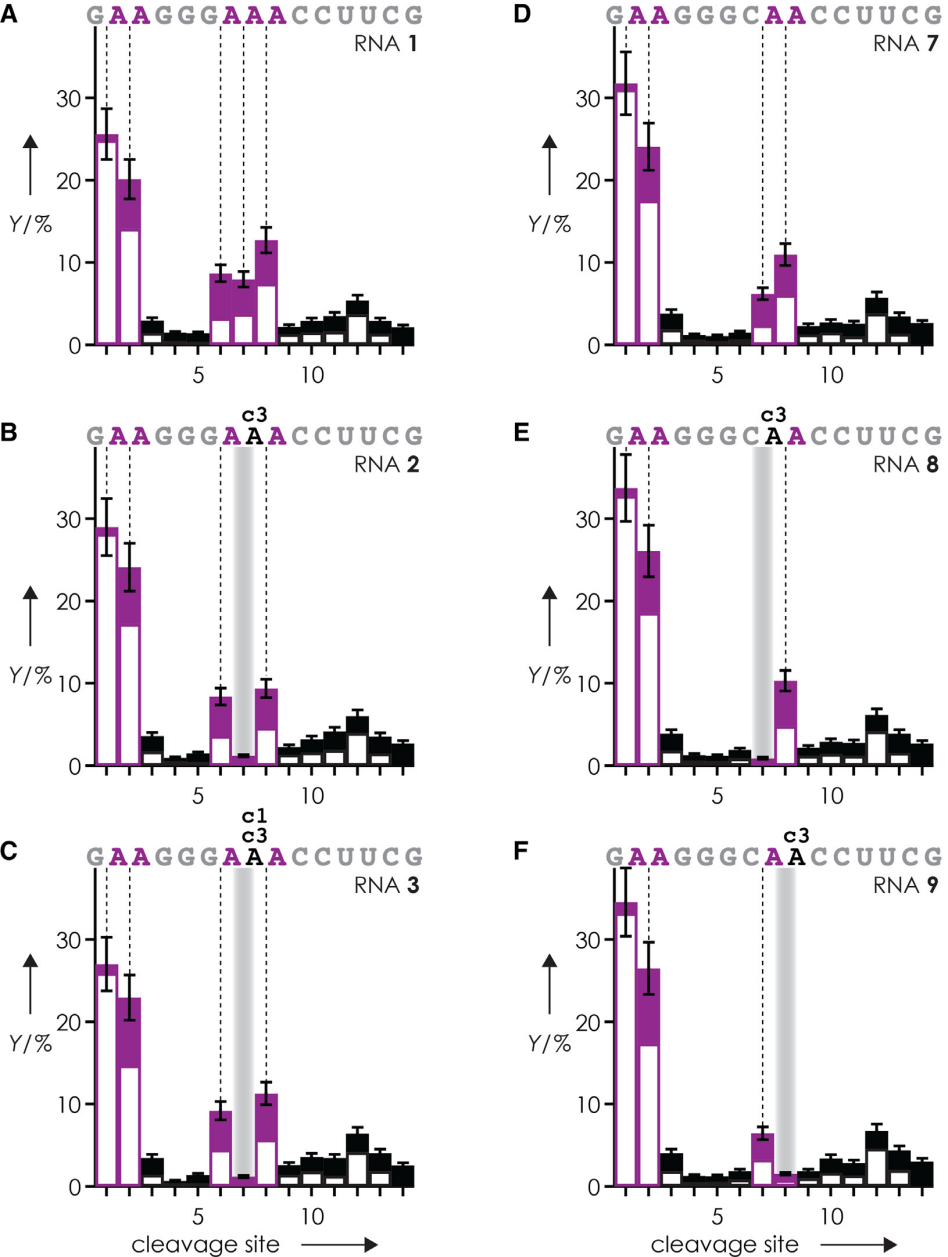
Yield *Y* of ***c*** (filled bars) and ***y*** (open bars) fragments (including those that showed H_2_O and/or nucleobase losses, normalized to the yield of all fragments from phosphodiester backbone bond cleavage) from CAD (42 eV laboratory frame collision energy) of (M+4H)^4+^ ions of (**A**) RNA **1**, (**B**) RNA **2**, (**C**) RNA **3**, (**D**) RNA **7**, (**E**) RNA **8**, and (**F**) RNA **9** versus cleavage site, those of fragments from phosphodiester backbone bond cleavage on the 5′-side of A and c^3^A are highlighted in purple.

Figure 1 shows spectra from CAD of (M+4H)^4+^ ions of RNA **1** (Table 1, unmodified RNA) and **2** (c^3^A at position 8). At first glance, the spectra are highly similar, with ***y***_14_^3+^, ***y***_13_^3+^ and (M+3H-guanine)^3+^ ions (the latter from loss of protonated guanine) as the most abundant products. The high yield of ***y***_14_^3+^ and ***y***_13_^3+^ fragments from phosphodiester backbone bond cleavage (Scheme 1D) at sites 1 and 2, on the 5′-side of A2 and A3, respectively, is consistent with previous observations of preferred phosphodiester backbone bond cleavage next to adenosine residues in CAD of (M+nH)^n+^ ions (36). The yield of the complementary ***c***_1_^+^ and ***c***_2_^+^ fragments was far lower (Figures 1 and 2A-B), presumably due to loss of protonated guanine that produces uncharged and thus undetectable ***c***_1_-guanine and ***c***_2_-guanine fragments; the nucleobase at the 5′-terminus is particularly labile in CAD of RNA (33,46). Moreover, the electrostatic ion transfer from the collision cell (used for CAD) to the FT-ICR cell (used for ion detection) caused some discrimination of ions (47,48) with lower mass-to-charge (*m/z*) ratio including ***c***_1_^+^ and ***c***_2_^+^. Detectability and ion discrimination was not an issue with the ***c*** and ***y*** fragments from backbone cleavage on the 5′-side of A7, A8 and A9 (sites 6–8) that all carried a net charge of 2+, including those that showed H_2_O and/or nucleobase loss (∼13%). Although more pronounced at sites 1 and 2, we therefore investigated the ‘A-effect’ at sites 6–8 by introduction of c^1^A, c^1,3^A, c^3^A and c^7^A (Table 1). As illustrated in Figure 1, the yield of ***c*** and ***y*** ions from cleavage at sites 6 (***c***_6_/***y***_9_, 5′-side of A7), 7 (***c***_7_/***y***_8_, 5′-side of A8) and 8 (***c***_8_/***y***_7_, 5′-side of A9) in CAD of (M+4H)^4+^ ions of RNA **1** was significantly higher than that from cleavage at sites 3–5 and 9–14 (Figures 1 and 2A). By contrast, CAD of (M+4H)^4+^ ions of RNA **2** with c^3^A instead of A at position 8 showed substantially decreased yields of complementary ***c***_7_ and ***y***_8_ fragments from phosphodiester backbone bond cleavage at site 7, on the 5′-side of c^3^A at position 8 (Figures 1 and 2B).

We have previously attributed the increased yield of ***c*** and ***y*** fragments from phosphodiester backbone bond cleavage on the 5′-side of adenosine residues in CAD of (M+nH)^n+^ ions of RNA to ionic hydrogen bonding between adenine protonated at N3 and a nonbridging oxygen of the adjacent phosphodiester group (5′-side) which was thought to facilitate nucleophilic attack of the 2′-oxygen onto the phosphorus (36). The lower yield of ***c***_7_ and ***y***_8_ fragments in CAD of (M+4H)^4+^ ions of RNAs **2** and **3** compared to that of RNA **1** (Figures 1 and 2A−C) strongly supports our hypothesis as N3 is not present and thus not available for protonation and ionic hydrogen bonding in c^3^A and c^1,3^A. Further, the yield of ***c*** and ***y*** fragments from phosphodiester backbone bond cleavage at sites 7 and 8 was, within error limits (Supplementary Figure S1), the same for RNA **1** (AAA at positions 7–9, Table 1) and RNA **7** (CAA at positions 7–9), and replacing A with c^3^A or c^1,3^A at positions 8 (RNA **2, 3, 8**) or 9 (RNA **9**) reduced the yield of ***c*** and ***y*** fragments from phosphodiester backbone bond cleavage at sites 7 and 8, respectively, without significantly affecting that of other sites (Figure 2). Replacing A with dA at position 8 (RNA **6**) and 9 (RNA **14**) did not significantly alter the fragmentation patterns except that no ***c*** and ***y*** fragments from cleavage on the 3′-side of dA (sites 8 and 9, respectively) were observed (Supplementary Figure S2), which is consistent with a self-cleavage mechanism that requires a 2′-OH group for nucleophilic attack on the phosphorus (36,49,50). These observations indicate that adenine protonated at N3 forms ionic hydrogen bonds predominantly with the phosphodiester group directly on its 5′-side instead of that on its 3′-side or other phosphodiester groups that are more remote in sequence.

**Figure 3.**
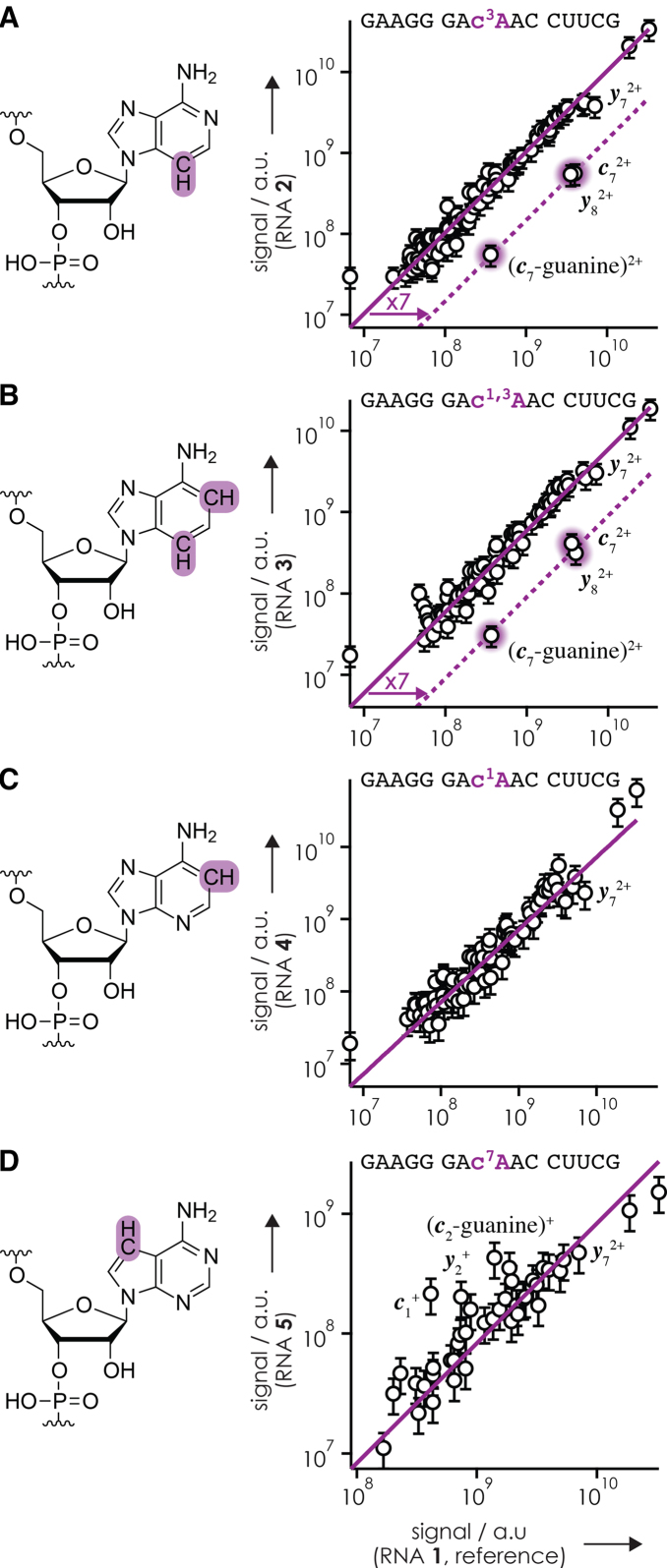
Signals of ***c*** and ***y*** fragments from CAD (42 eV laboratory frame collision energy) of (M+4H)^4+^ ions of (**A**) RNA **2** (c^3^A at position 8), (**B**) RNA **3** (c^1,3^A at position 8), (**C**) RNA **4** (c^1^A at position 8) and (**D**) RNA **5** (c^7^A at position 8), versus those of the unmodified reference RNA **1** (AAA at positions 7–9). Signals of fragments that were significantly higher in the spectra from CAD of (M+4H)^4+^ ions of the reference RNA **1** compared to those of the RNA under investigation are highlighted by purple shading.

**Figure 4.**
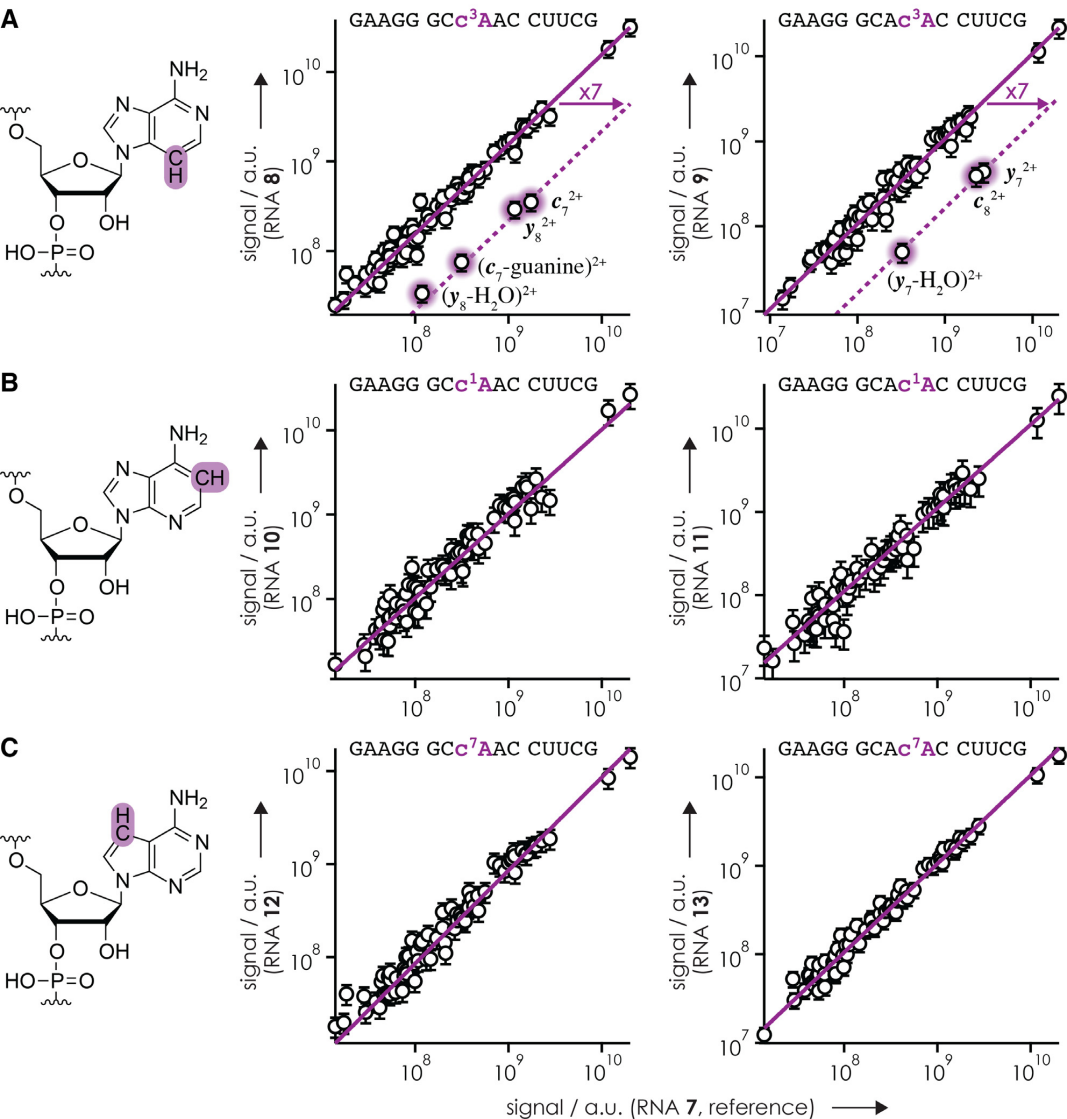
Signals of ***c*** and ***y*** fragments from CAD (42 eV laboratory frame collision energy) of (M+4H)^4+^ ions of (**A**) c^3^A-modified RNA **8** (position 8) and **9** (position 9), (**B**) c^1^A-modified RNA **10** (position 8) and **11** (position 9) and (**C**) c^7^A-modified RNA **12** (position 8) and **13** (position 9), versus those of the unmodified reference RNA **7** (CAA at positions 7–9).

To evaluate whether or not the slightly lower yield of fragments from cleavage at sites 6 (***c***_6_^2+^) and 8 (***c***_8_^2+^,***y***_7_^2+^) in CAD of RNA **2** (c^3^A at position 8, Figure 1) compared to RNA **1** (A at position 8) was significant, we plotted the signals of products from CAD of RNA **2**–**5** versus those of the reference RNA **1** (Figure 3). In all cases, the data correlated linearly, but for RNA **2** and **3, *c***_7_^2+^, (***c***_7_-guanine)^2+^and ***y***_8_^2+^ from backbone cleavage at site 8 clearly stood out. The Pearson correlation coefficients for the linear fits in Figure 3 were 0.99 (RNA **2**; ***c***_7_^2+^, (***c***_7_-guanine)^2+^, and ***y***_8_^2+^ excluded), 0.96 (RNA **3**; ***c***_7_^2+^, (***c***_7_-guanine)^2+^, and ***y***_8_^2+^ excluded), 0.84 (RNA **4**), and 0.82 (RNA **5**). For RNA **2**, the signal of ***y***_7_^2+^, but not those of ***c***_6_^2+^ and ***c***_8_^2+^ (the complement of ***y***_7_^2+^), deviated from the linear fit (Figure 3A). For RNA **3**, no significant deviation of the ***y***_7_^2+^, ***c***_6_^2+^ or ***c***_8_^2+^ signals was found (Figure 3B). Moreover, ***y***_7_^2+^ did not deviate from the linear correlation of ***c*** and ***y*** signals from CAD of RNA **8** versus those of RNA **7** (Figure 4A), so we conclude that the slight deviation of ***y***_7_^2+^ in Figure 3A is not significant. The largest scatter in signal was observed for RNA **5** versus RNA **1** (Figure 3D), which can be attributed to a smaller number of ions in the CAD experiment with RNA **5** and correspondingly decreased statistics.

Importantly, Figures 3 and 4 show that only c^3^A and c^1,3^A, but not c^1^A or c^7^A, inhibited preferred backbone cleavage on the 5′-side of adenosine residues, which further substantiates our hypothesis of ionic hydrogen bonding between adenine protonated at N3, but not N1 or N7, and an oxygen of the adjacent phosphodiester group (36). The ∼7-fold decrease in signal of ***c***_7_^2+^, (***c***_7_-guanine)^2+^ and ***y***_8_^2+^ in CAD of RNA **2** and **3** (Figure 3A and B) quantifies the effect of A at site 7 of RNA **1**. Likewise, c^3^A, but not c^1^A or c^7^A, inhibited the effect of A at sites 7 and 8 of RNA **7** as evidenced by an ∼7-fold reduction of the ***c***_7_^2+^, (***c***_7_-guanine)^2+^, ***y***_8_^2+^, (***y***_8_-H_2_O)^2+^ and ***c***_8_^2+^, ***y***_7_^2+^, (***y***_7_-H_2_O)^2+^ signals in CAD of RNA **8** and **9**, respectively (Figure 4). The signals of products from CAD of RNA **7** (CAA at positions 7–9) also correlated linearly (Pearson coefficient of 0.995) with those of RNA **1** (AAA at positions 7–9), except for ***c***_6_^2+^, (***c***_6_-guanine)^2+^ and ***y***_9_^2+^ from backbone cleavage at site 6, which again deviated by a factor of ∼7 (Supplementary Figure S3). Thus for the 15 nt RNAs studied here, the ‘A-effect’ in CAD at 42 eV laboratory frame collision energy of (M+4H)^4+^ ions electrosprayed from solutions at pH 6.8 is uniformly ∼7 at sites 7–9.

**Scheme 2.**
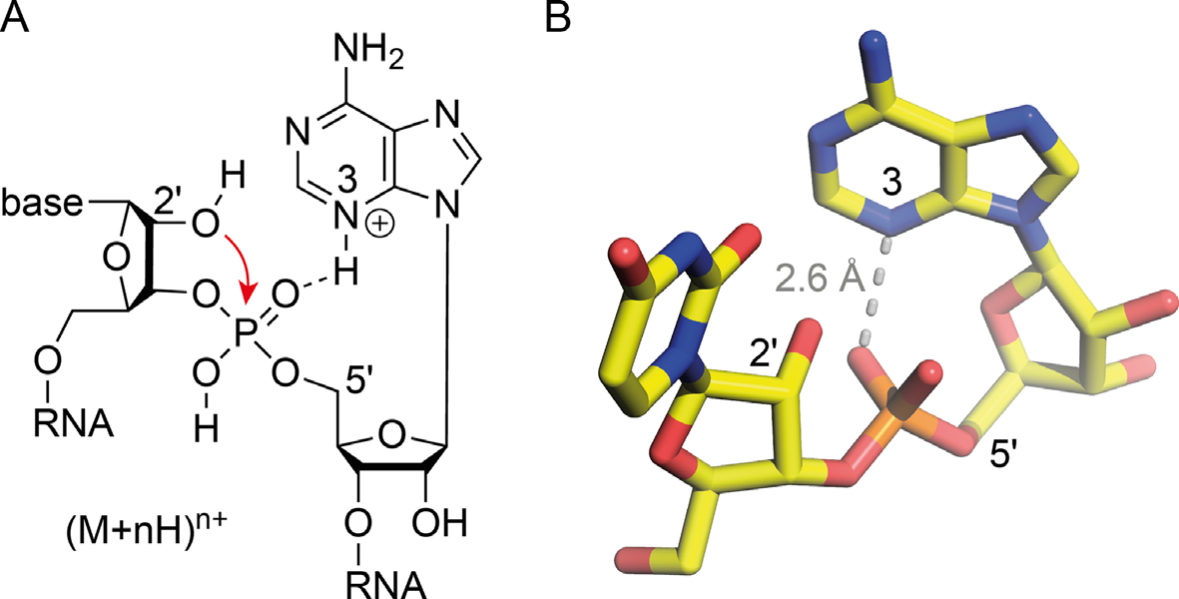
(**A**) Proposed mechanism for phosphodiester backbone bond cleavage in CAD of (M+nH)^n+^ ions of RNA in which nucleophilic attack of the 2′-OH group on the phosphorus (arrow) is facilitated by ionic hydrogen bonding (dashed line) between A protonated at N3 and the phosphodiester moiety and (**B**) corresponding dinucleotide model of UA.

The effect of adenosine residues on the yield of ***c*** and ***y*** fragments from CAD of gaseous RNA (M+nH)^n+^ ions observed here, and the lack of it when A is replaced by c^3^A or c^1,3^A but not c^1^A or c^7^A (Figures 3 and 4), implies adenine protonation at N3, in agreement with data from hydrogen/deuterium exchange (51) and a combined experimental and theoretical study of adenosine-5′-monophosphoric acid (M+H)^+^ ions (52). The increased proton affinity of N3 over that of N1 and N7 in adenosine-5′-monophosphoric acid (M+H)^+^ ions results from stabilization (by >35 kJ/mol) of a *syn*, C2′-endo conformation in which the protonated N3 forms an ionic hydrogen bond with a nonbridging oxygen of the uncharged monophosphoester moiety (N3H^+^···O = P motif) (52). This is the very same motif that we hold responsible for facilitating nucleophilic attack of the 2′-OH group on the phosphorus in the course of phosphodiester backbone bond cleavage by CAD of (M+nH)^n+^ ions of RNA (Scheme 2), although we cannot exclude the possibility of ionic hydrogen bonding to a bridging instead of a non-bridging oxygen of the uncharged phosphodiester moiety. In the absence of a phosphoester moiety, hydrogen bonding between adenine and the ribose moiety gives rise to preferred protonation (>25 kJ/mol stabilization) of N3 over N1 and N7 in (M+H)^+^ ions of adenosine (53), whereas adenine by itself is preferentially protonated at N1 both in the gas phase (54,55) and in solution (14). Apparently, protonated adenine has a high intrinsic propensity to form ionic hydrogen bonds with oxygen in gaseous nucleoside, nucleotide and RNA (M+nH)^n+^ ions.

In RNA structures from NMR spectroscopy and X-ray crystallography, protonated nucleobases can be found in non-Watson–Crick base pairs (6,56–58) and in interactions with phosphates (10,59). Among all non-base-pair interactions, nucleobase–phosphate interactions have been considered the most important ones as most of them are phylogenetically conserved and ubiquitous (60,61). The majority of these interactions supposedly involve uncharged nucleobases and phosphates. However, little is known to date about the role of charged nucleobases (6,62) and their interactions with phosphates of the RNA backbone. RNA nucleobases are generally not protonated (or deprotonated) in solutions at neutral pH (63–65), but their p*K* values can shift toward neutrality (59,62,66–73) in the microenvironment of distinct RNA folds that afford favorable hydrogen bonding networks and electrostatic stabilization (6). As pointed out by Honig and coworkers, the favorable interactions of a protonated base with negatively charged phosphate groups (base-phosphate interactions) will always favor a shift to higher p*K* values (59). Regarding adenine, the exact site of protonation can be different in different RNA scaffolds (6). For example, adenine protonation at N1 is highly preferred over protonation at N3 in parallel duplex formation of A-tracts via trans Hoogsteen–Hoogsteen A•A base pairing (74). Adenine protonation at N1 is also critical to ligand recognition in riboswitches; by using NMR spectroscopy, Wöhnert and coworkers demonstrated that the c-GAMP riboswitch binds to c-di-GMP via a stably N1-protonated adenine in the binding pocket, even at pH values well above neutrality (75). By contrast, N3 has been identified for adenine protonation to play a critical role in the catalytic reaction of the twister ribozymes (3,13,76–79). More specifically, crystal structures of the twister ribozymes (13,76) showed that the adenine 3′ to the scissile phosphate is in *syn* conformation, with its N3 atom in vicinity to the ribose 5′-O leaving group (Scheme 1B). An NMR study revealed that the p*K* of this very adenine (A6) is shifted toward neutrality, which is consistent with its proposed role in general acid base catalysis by acting as proton donor in the twister cleavage reaction (3,13,76). The conformation of U5-A6 in the above structures, with *syn* orientation of A6, closely resembles that found for gaseous (M+H)^+^ ions of adenosine monophosphoric acid (52) and the here proposed conformation of (M+nH)^n+^ ions of RNA illustrated in Scheme 2.

Secondary structure calculations (http://rna.tbi.univie.ac.at) (80) predict that in aqueous solution at ambient temperature, RNAs **1** and **7** should have highly similar hairpin structures of similar stability. However, the (M+4H)^4+^ RNA ions studied here were electrosprayed from denaturing solutions in 50% CH_3_OH to prevent the formation of secondary structure (81,82). Although we cannot exclude the possibility that a small fraction of the (M+4H)^4+^ ions of RNAs **1** and **7** can exist as hairpin conformations, the pronounced effect of A at cleavage sites 1 and 2 (Figure 2) suggests that the majority of ions have extended, unpaired conformations because Watson-Crick base pairing of adenine in the stem of a putative hairpin (A2-U13, A3-U12) and adenine *syn* conformations (36) are mutually exclusive. Further, the fact that we found no evidence for ionic hydrogen bonds between protonated adenine and phosphodiester groups that are more remote in sequence suggests that the majority of (M+4H)^4+^ RNA ions have largely extended structures. RNA structures are generally not the same in solution and the gas phase (83,84), but inter- and intramolecular interactions that involve charged sites can be preserved during and after transfer from solution into the gas phase by ESI (28,29,85–88). We were thus wondering if the pH of the RNA solution had any effect on adenine protonation and its interactions with adjacent phosphodiester groups (the here proposed intramolecular N3H^+^···O = P motif), and if this would be reflected in the ESI or CAD spectra.

**Figure 5.**
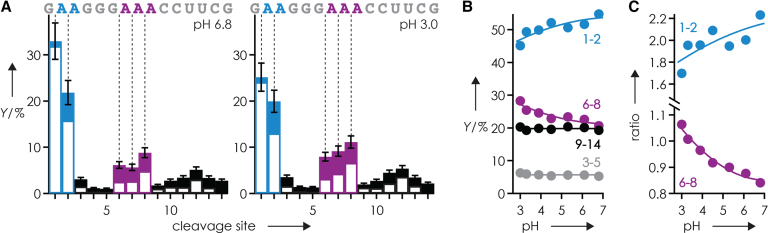
(**A**) Site-specific yield of ***c*** and ***y*** fragments from CAD (42 eV laboratory frame collision energy) of (M+4H)^4+^ ions of RNA **1** electrosprayed from solutions at pH 6.8 and 3.0, (**B**) added yields for sites 1–2 (blue), 3–5 (gray), 6–8 (purple) and 9–14 (black) and (**C**) added yields for sites 1–2 (blue) and 6–8 (purple) divided by the added yields from sites 3–5 and 9–14 versus solution pH.

Lowering the pH of the ESI solution from 6.8 to 3.0 by addition of acetic acid did not significantly affect the relative abundances of (M+nH)^n+^ ions of RNA **1**, i.e. ∼95% (M+4H)^4+^ and ∼5% (M+3H)^3+^ ions, but their overall abundance substantially decreased with decreasing pH (Supplementary Figure S4). This observation is consistent with previous studies in which the ionization efficiency in ESI of proteins was related to pI and solution pH (89). Nevertheless, by adjustment of the time for ion accumulation in the mass spectrometer (34) such that similar numbers of (M+4H)^4+^ ions of RNA **1** were available for CAD in each experiment, it was possible to study the effect of pH on the yield of ***c*** and ***y*** fragments without any bias that could result from limited signal-to-noise ratio. Figure 5A shows that the yield of ***c*** and ***y*** fragments from phosphodiester backbone bond cleavage at sites 3–5 and 9–14 in CAD of (M+4H)^4+^ ions of RNA **1** electrosprayed from solutions at pH 6.8 and 3.0 was similar, but that the yield of ***c*** and ***y*** fragments from cleavage at sites 1–2 and 6–8 was affected by pH. Because the variation in signal of ***y***_13_^3+^ and ***y***_14_^3+^ from cleavage at sites 1–2 was stronger than for other fragments as a result of Coulombic interactions (Supplementary Figure S1), we also plotted the yield for sites 1–2 and 6–8 divided by that from sites 3–5 and 9–14, which further confirmed the effect of solution pH (Figure 5C). The effect of pH was smaller than the effect of A though as preferential phosphodiester backbone bond cleavage next to adenine (sites 1, 2, 6, 7 and 8) was observed over the entire pH range studied (Figure 5).

**Figure 6.**
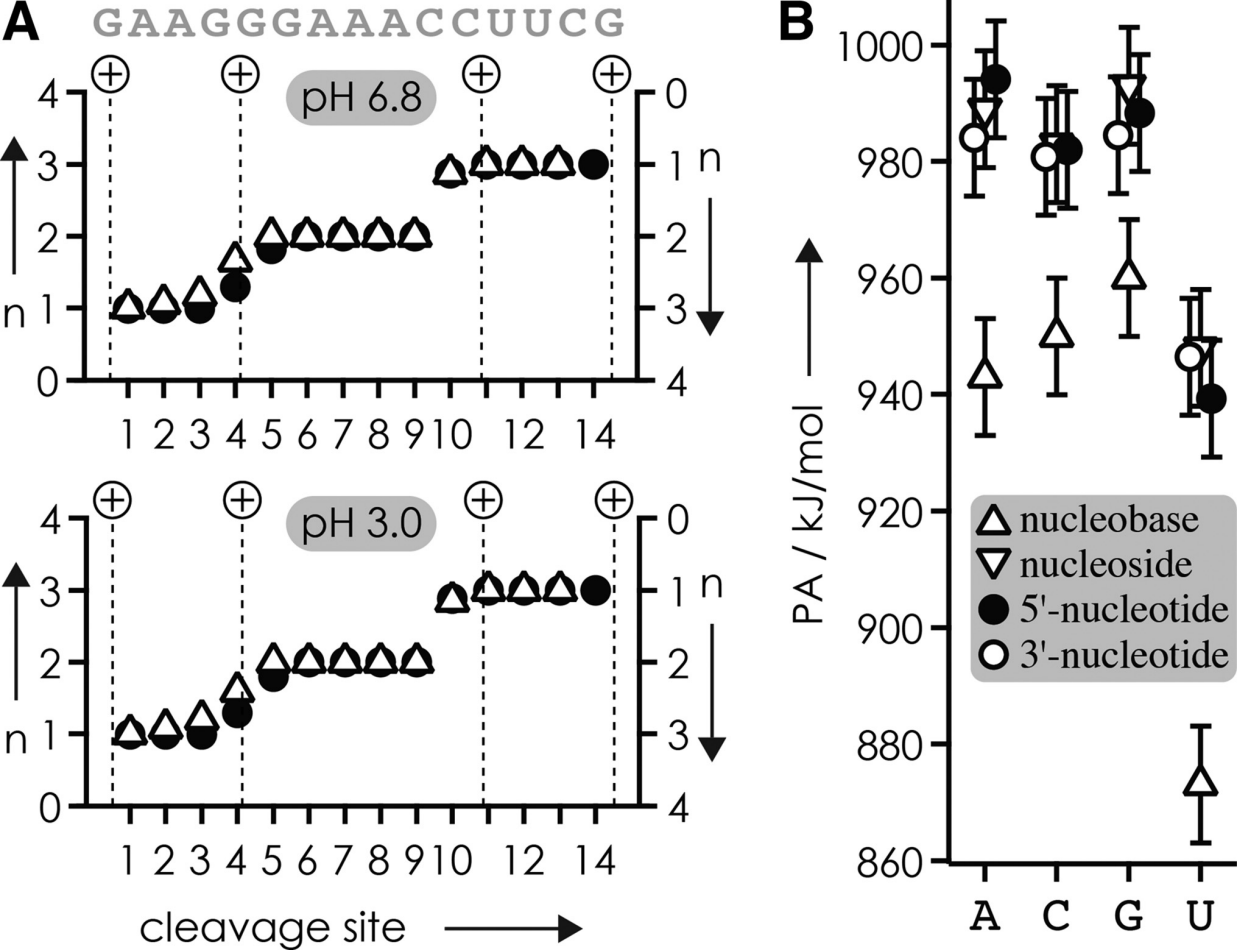
(**A**) Average charge n of ***c*** (circles, left axis) and ***y*** (triangles, right axis) fragments from CAD (42 eV laboratory frame collision energy) of (M+4H)^4+^ ions of RNA **1** electrosprayed from solutions at pH 6.8 and 3.0, dashed lines indicate calculated charge locations according to Coulombic repulsion in extended RNA structures (36); (**B**) proton affinities of nucleobases (90), nucleosides (90) and nucleotides with the phosphoester group in the 5′- or 3′-position (91), all with errors of ±10 kJ/mol (99).

With the number of protons and the net positive charge being the same in all (M+4H)^4+^ ions of RNA **1**, the data in Figure 5 imply that the distribution of protons within ions electrosprayed from solutions at different pH must be different. More specifically, the increase in yield of ***c*** and ***y*** fragments from cleavage at sites 6–8 with decreasing pH, and the corresponding decrease in yield of ***c*** and ***y*** fragments from cleavage at sites 1–2 (Figure 5 B and C), indicate that protonation of A7, A8, and A9 increases at the expense of A2 and A3. However, the protonation patterns indicated by the site-specific ***c*** and ***y*** fragment yields (Figure 5A), with preferential protonation of A residues (Scheme 2), are not preserved in the ***c*** and ***y*** fragments as their average charge values were virtually the same at pH 3.0 and 6.8 (Figure 6A). Moreover, we found highly similar average charge values for ***c*** and ***y*** fragments from CAD of (M+4H)^4+^ ions of all other RNAs studied here (Table 1; Supplementary Figures S5 and 6).

The average charge values of ***c*** and ***y*** fragments from CAD of (M+4H)^4+^ ions of RNAs **1**–**14** (Figure 6A; Supplementary Figures S5 and 6), and those from CAD of (M+nH)^n+^ and (M-nH)^n−^ ions of 14 different, 13–22 nt RNAs in our previous study (36), all indicate charge locations according to Coulombic repulsion in extended RNA structures regardless of solution pH and the extent of phosphodiester backbone bond cleavage. We have rationalized the seeming discrepancy in charge locations required for preferred phosphodiester backbone bond cleavage and those resulting from Coulombic repulsion by a stepwise mechanism for RNA dissociation into ***c*** and ***y*** fragments by CAD (36). In the first step at low energy, a pentacoordinate oxyphosphorane intermediate is formed by nucleophilic attack of a ribose 2′-OH group on the adjacent phosphorus, which can be facilitated by ionic hydrogen bonding as illustrated in Scheme 2. In the next step at elevated ion internal energy, the breaking of hydrogen bonds leads to full extension of the RNA (M+nH)^n+^ ion structure and subsequent intramolecular proton transfer according to simple Coulombic repulsion. In the last step at even higher ion internal energy, the intermediate dissociates into ***c*** and complementary ***y*** fragments by cleavage of the phosphodiester backbone bond. The data in Figure 5 strongly support the order of events in our proposed mechanism. First, the proton affinities (PA) of A, C and G nucleosides and nucleotides (90,91) are the same within error limits (Figure 6B), such that intramolecular proton transfer before nucleophilic attack would not favor protonation of A. Second, Coulombic repulsion would transfer protons away from the center residues (A7, A8 and A9) and increase protonation of residues at and near each terminus, including A2 and A3 (36). Both proton affinity and Coulombic repulsion oppose the observed effect of decreasing pH, that is, intramolecular proton redistribution away from A2 and A3, and to A7, A8, and A9 (Figure 5). At last, if the site-specific extent of protonation of RNA **1** were the same in solutions at pH 3.0 and 6.8, there is no reason for any differences in intramolecular proton transfer in the corresponding (M+4H)^4+^ ions from ESI. We conclude that the effect of pH illustrated in Figure 5 must result from different protonation patterns in solution that are at least in part preserved during and after transfer into the gas phase, thereby affecting nucleophilic attack of ribose 2′-OH groups on adjacent phosphorus atoms and dissociation of the gaseous (M+4H)^4+^ ions into ***c*** and ***y*** fragments by CAD (Scheme 2).

The p*K* values for protonation of the nucleobase in C, A and G ribonucleosides in aqueous solution are ∼4.2 (N3), ∼3.6 (N1) and ∼1.6 (N7), respectively (63,92); that of U is far lower. The p*K* values for nucleobase protonation in ribonucleoside 3′,5′-bis-ethylphosphates are ∼4.2 for C and ∼3.7 for A, and differ from those of deoxyribonucleoside 3′,5′-bis-ethylphosphates by only ∼0.1 p*K* units (65). Nucleobase protonation by gradual acidification of solutions of DNA followed the order C before A before G (94,95), and for 2–13 nt DNA, the p*K* values of both A and C increased with increasing number of residues (95). Further, experimental studies on a number of ribozymes and riboswitches have shown that the p*K* values of both A and C can shift toward neutrality (93). This raises the question why only protonation of A, but not C, facilitates nucleophilic attack of ribose 2′-OH groups on adjacent phosphorus atoms and dissociation of the RNA (M+nH)^n+^ ions into ***c*** and ***y*** fragments. The site of protonation of C in both RNA and DNA in solution is its N3 atom (93,96), whereas for gaseous (deoxy)ribonucleoside and -nucleotide (M+H)^+^ ions, both N3 and O2 protonated conformers coexist in the gas phase (97,98). In both the N3 and the O2 nucleotide conformers, the nucleobase is in *anti* orientation such that only a relatively weak C6H···O = P but no ionic N3H^+^···O = P or O2H^+^···O = P hydrogen bonds are formed (98). The lack of any appreciable effect of C on phosphodiester backbone bond cleavage in CAD of (M+nH)^n+^ ions of RNA can thus be attributed to the lack of a stabilizing charge in the C6H···O = P interaction (99).

## CONCLUSION

Our experimental study of 15 nt RNAs and their deaza-derivatives with site-specific incorporation of c^1^A, c^1,3^A, c^3^A, and c^7^A residues provides straightforward evidence that adenine protonated at N3 but not N1 or N7 forms ionic hydrogen bonds with an oxygen of the adjacent phosphodiester group in gaseous (M+nH)^n+^ ions of RNA, thereby facilitating nucleophilic attack of the 2′-oxygen onto the phosphorus on its 5′-side and dissociation into ***c*** and complementary ***y*** fragments. CAD of (M+4H)^4+^ ions electrosprayed from solutions at different pH shows that the protonation patterns in solution are at least in part preserved after transfer into the gas phase and during nucleophilic attack. Gas phase basicity and p*K* values suggest that protonation of A at neutral pH is energetically favorable in RNA structures that allow for ionic hydrogen bonding between adenine and the phosphodiester group on its 5′-side. This interaction involves protonation at N3 instead of N1 and *syn* conformation of adenine, which is generally incompatible with typical RNA secondary structure elements based on nucleobase pairing and stacking interactions, i.e. helix structures. However, in RNA loop and bulge regions that frequently act as binding and/or active sites, the intrinsic preference of adenine for interactions with the adjacent phosphodiester moiety and its contribution to RNA function may be more prevalent than previously anticipated.

## Supplementary Material

gkz574_Supplemental_File
