# Supplementary material for: The effect of adenine protonation on RNA phosphodiester backbone bond cleavage elucidated by deaza-nucleobase modifications and mass spectrometry
Source: Nucleic Acids Res. 2019 Jul 5;47(14):7223–34. doi: 10.1093/nar/gkz574 (PMC6698743; doi:10.1093/nar/gkz574)
Supplement: gkz574_Supplemental_File [file gkz574_supplemental_file.pdf]

SUPPLEMENTARY DATA

The effect of adenine protonation on RNA phosphodiester backbone bond cleavage elucidated by deaza-nucleobase modifications and mass spectrometry

E. Fuchs, C. Falschlunger, R. Micura, and K. Breuker

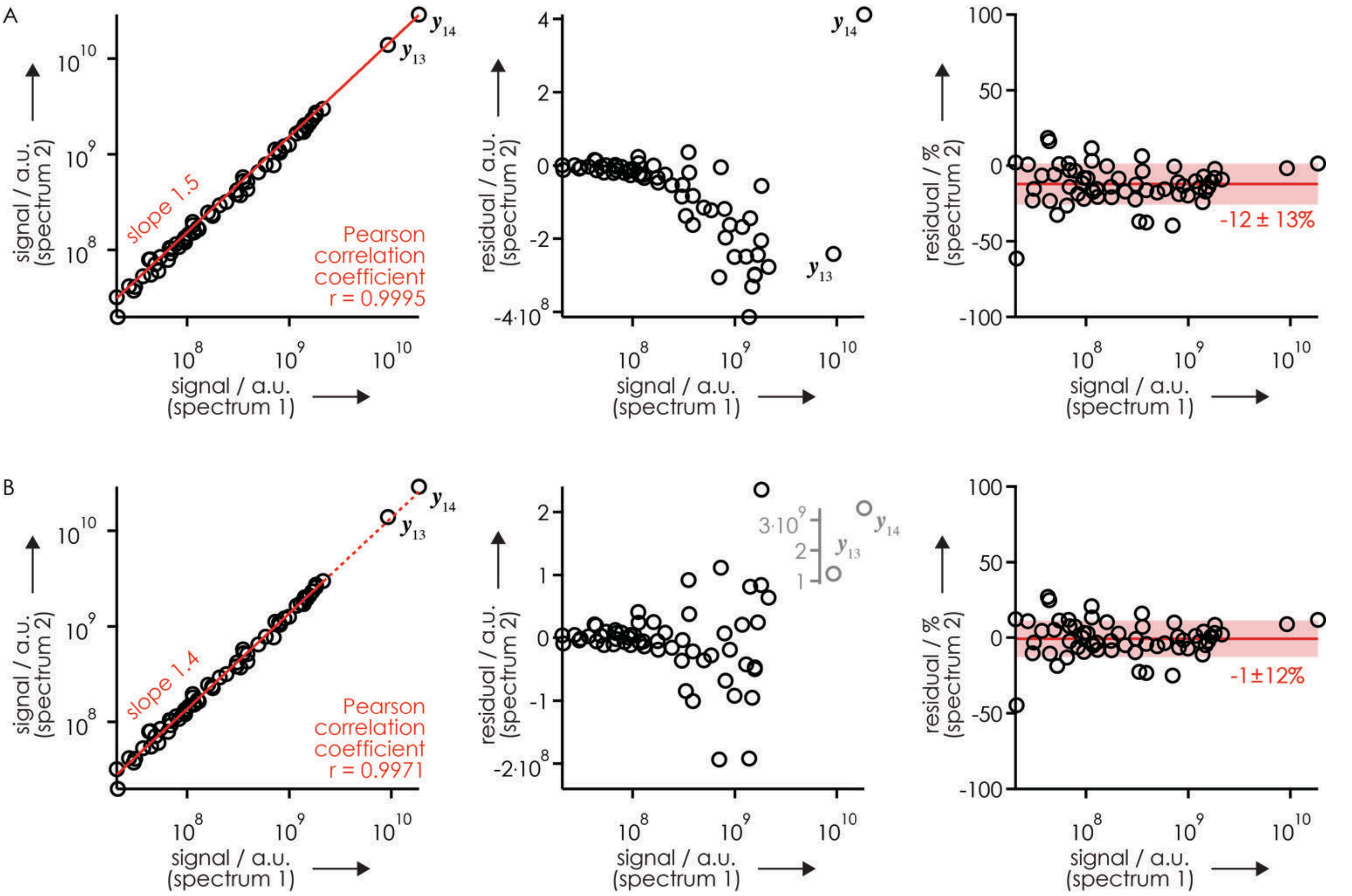

Figure S1. Error analysis using two different spectra from CAD (42 eV laboratory frame collision energy) of $(M+4H)^{4+}$ ions of RNA **1**: Signals (in arbitrary units) of c and y fragments from spectrum 2 plotted versus those from spectrum 1 with linear fits (left), residuals (center), and residuals in % of signals (right); A) the linear fit (zero intercept) of all signals reveals a systematic bias that can be attributed to Coulombic interactions (space charge of trapped ions, whose number was higher in the experiment for spectrum 2 compared to that for spectrum 1 by a factor of ~1.4); B) excluding signals of the highly abundant y_{13}^{3+} and y_{14}^{3+} ions from the linear fit largely eliminates the bias and allows for calculation of a standard deviation of ±12% for the data in Figures 2, 5A, and S2. Error analysis for the data in Figures 3, 4, and S3 was performed in a similar fashion by linear least-square fitting of signals in sample versus reference spectra.

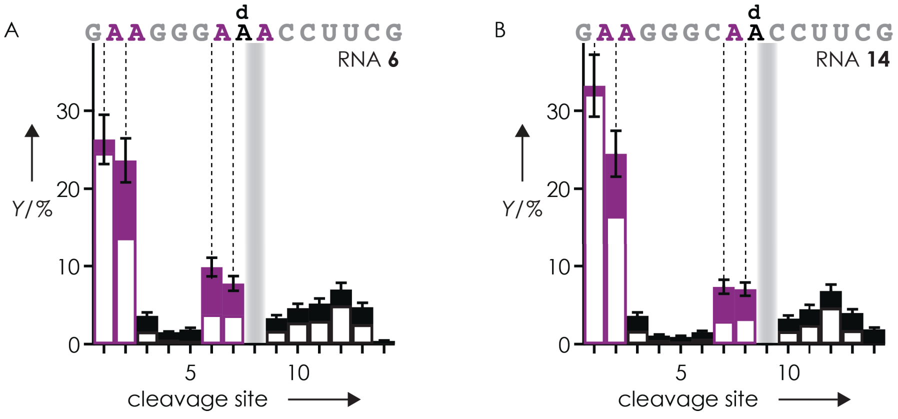

Figure S2. Yield of c (filled bars) and y (open bars) fragments (including those that showed H_2O and/or nucleobase losses, normalized to the yield of all fragments from phosphodiester backbone bond cleavage) from CAD (42 eV laboratory frame collision energy) of $(M+4H)^{4+}$ ions of A) RNA **6** and B) RNA **14** versus cleavage site, those of fragments from phosphodiester backbone bond cleavage on the 5'-side of A are highlighted in purple.

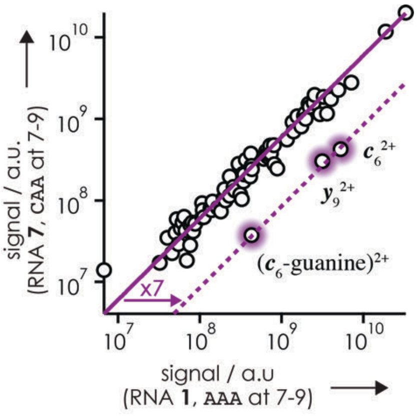

Figure S3. Signals of c and y fragments from CAD (42 eV laboratory frame collision energy) of $(M+4H)^{4+}$ ions of RNA **7** (`CAA` at positions 7-9) versus those of RNA **1** (`AAA` at positions 7-9)**.**

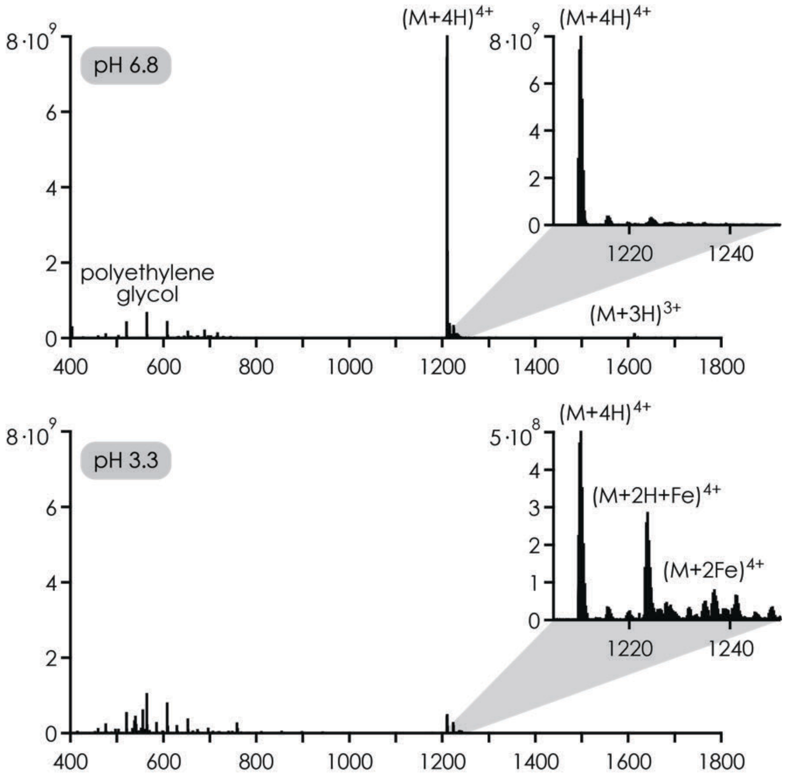

Figure S4. ESI spectra of 1 μM RNA **1** in 1:1 H_2O/CH_3OH with 20 mM ammonium acetate at pH 6.8 and 3.3, adjusted by the addition of acetic acid (which also releases some Fe^{2+} from the metallic ESI emitter).

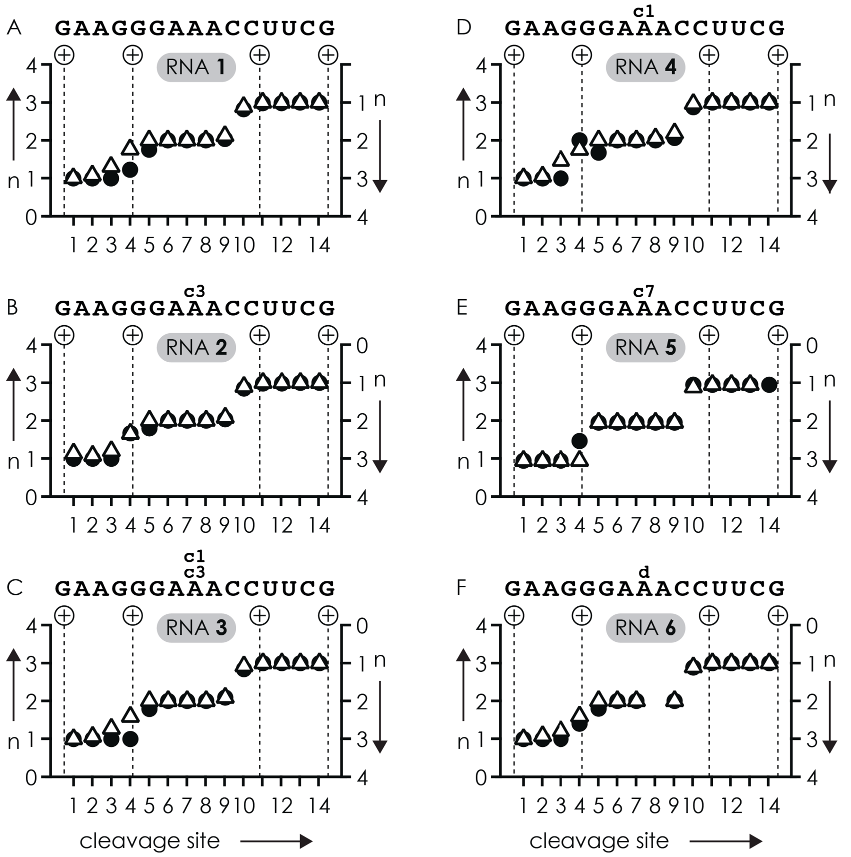

Figure S5. Average charge of *c* (left axis) and *y* (right axis) fragments from CAD (42 eV laboratory frame collision energy) of $(M+4H)^{4+}$ ions of A) RNA **1**, B) RNA **2**, C) RNA **3**, D) RNA **4**, E) RNA **5**, and F) RNA **6** electrosprayed from solutions at pH 6.8, dashed lines indicate calculated charge locations according to Coulombic repulsion in extended RNA structures.

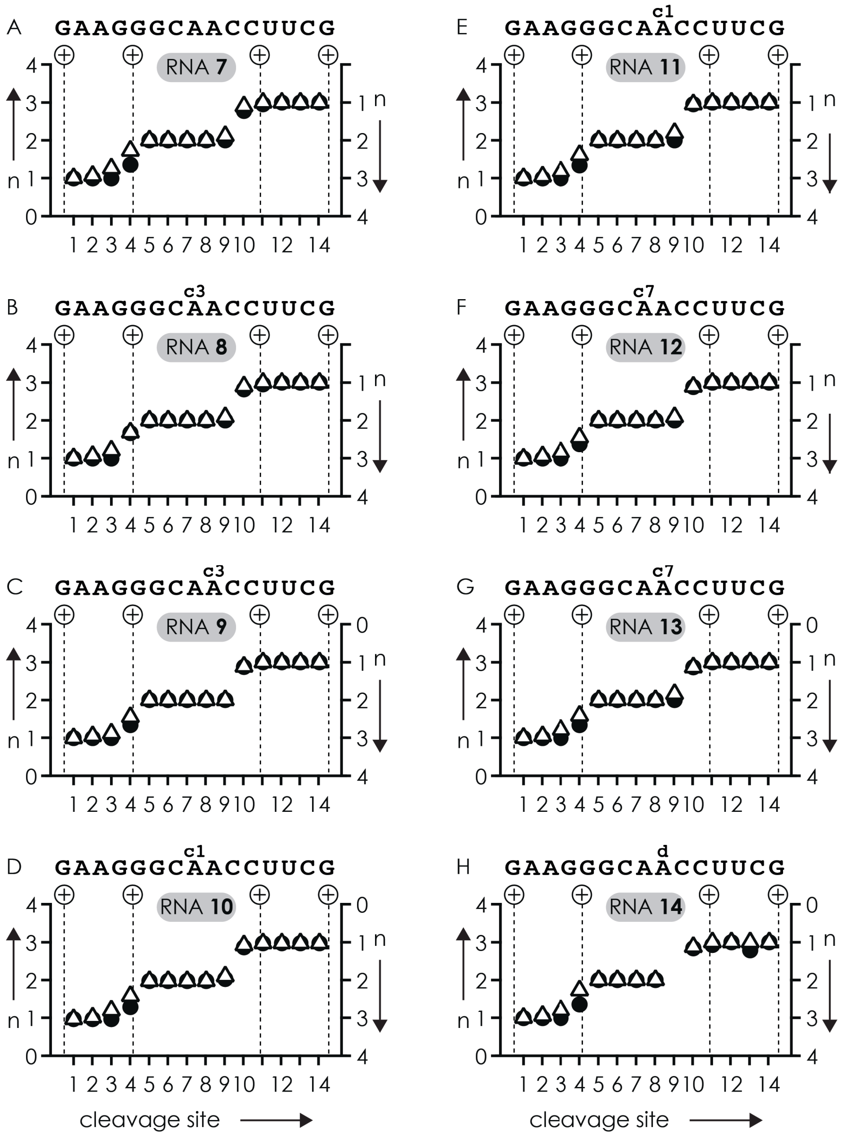

Figure S6. Average charge of *c* (left axis) and *y* (right axis) fragments from CAD (42 eV laboratory frame collision energy) of $(M+4H)^{4+}$ ions of A) RNA **7**, B) RNA **8**, C) RNA **9**, D) RNA **10**, E) RNA **11**, F) RNA **12**, G) RNA **13**, and H) RNA **14** electrosprayed from solutions at pH 6.8, dashed lines indicate calculated charge locations according to Coulombic repulsion in extended RNA structures
